# Cytokine release syndrome in a patient with colorectal cancer after vaccination with BNT162b2

**DOI:** 10.1038/s41591-021-01387-6

**Published:** 2021-05-26

**Authors:** Lewis Au, Annika Fendler, Scott T. C. Shepherd, Karolina Rzeniewicz, Maddalena Cerrone, Fiona Byrne, Eleanor Carlyle, Kim Edmonds, Lyra Del Rosario, John Shon, Winston A. Haynes, Barry Ward, Ben Shum, William Gordon, Camille L. Gerard, Wenyi Xie, Nalinie Joharatnam-Hogan, Kate Young, Lisa Pickering, Andrew J. S. Furness, James Larkin, Ruth Harvey, George Kassiotis, Sonia Gandhi, Crick COVID-19 Consortium, George Kassiotis, Sonia Gandhi, Charles Swanton, Charles Swanton, Charlotte Fribbens, Katalin A. Wilkinson, Robert J. Wilkinson, David K. Lau, Susana Banerjee, Naureen Starling, Ian Chau, CAPTURE Consortium, Lewis Au, Annika Fendler, Scott T. C. Shepherd, Fiona Byrne, Ben Shum, Camille Gerard, Kate Young, Lisa Pickering, Andrew J. S. Furness, James Larkin, George Kassiotis, Katalin A. Wilkinson, Robert J. Wilkinson, Susana Banerjee, Naureen Starling, Ian Chau, Samra Turajlic, Samra Turajlic

**Affiliations:** 1 Cancer Dynamics Laboratory, The Francis Crick Institute, London, UK; 2 Skin and Renal Units, The Royal Marsden NHS Foundation Trust, London, UK; 3 Tuberculosis Laboratory, The Francis Crick Institute, London, UK; 4 Department of Infectious Disease, Imperial College London, London, UK; 5 Serimmune, Inc., Goleta, CA USA; 6 Precision Oncology Center, Lausanne University Hospital (CHUV), Lausanne, Switzerland; 7 Worldwide Influenza Centre, The Francis Crick Institute, London, UK; 8 Retroviral Immunology Laboratory, The Francis Crick Institute, London, UK; 9 Neurodegeneration Biology Laboratory, The Francis Crick Institute, London, UK; 10 UCL Queen Square Institute of Neurology, Queen Square, London, UK; 11 Cancer Evolution and Genome Instability Laboratory, The Francis Crick Institute, London, UK; 12 Acute Oncology Service, The Royal Marsden NHS Foundation Trust, London, UK; 13 Gastrointestinal and Lymphoma Unit, The Royal Marsden NHS Foundation Trust, Sutton, UK; 14 Gynaecology Unit, The Royal Marsden NHS Foundation Trust and Institute of Cancer Research, London, UK

**Keywords:** RNA vaccines, Cancer immunotherapy, Colon cancer

## Abstract

Patients with cancer are currently prioritized in coronavirus disease 2019 (COVID-19) vaccination programs globally, which includes administration of mRNA vaccines. Cytokine release syndrome (CRS) has not been reported with mRNA vaccines and is an extremely rare immune-related adverse event of immune checkpoint inhibitors. We present a case of CRS that occurred 5 d after vaccination with BTN162b2 (tozinameran)—the Pfizer-BioNTech mRNA COVID-19 vaccine—in a patient with colorectal cancer on long-standing anti-PD-1 monotherapy. The CRS was evidenced by raised inflammatory markers, thrombocytopenia, elevated cytokine levels (IFN-γ/IL-2R/IL-18/IL-16/IL-10) and steroid responsiveness. The close temporal association of vaccination and diagnosis of CRS in this case suggests that CRS was a vaccine-related adverse event; with anti-PD1 blockade as a potential contributor. Overall, further prospective pharmacovigillence data are needed in patients with cancer, but the benefit–risk profile remains strongly in favor of COVID-19 vaccination in this population.

## Abstract (web summary)

A rare case of cytokine release syndrome in a patient on anti-PD-1 blockade that was likely related to BNT162b2 vaccination supports prospective monitoring of patients with cancer after COVID-19 vaccine administration.

## Main

CRS/cytokine storm is a systemic inflammatory response, characterized by excessive cytokine release (that is, elevated INF-γ, IL-6, IL-10 and IL-2R)^1^. CRS might develop after infection (including COVID-19) or due to iatrogenic causes, most notably chimeric antigen receptor T cell (CAR-T) therapy and, less frequently, cytotoxic chemotherapy or stem cell transplantation^1–3^. Extremely rarely, it occurs after immune checkpoint inhibitor (ICI) therapy^1,4^, and, to our knowledge, it has not been reported after administration of any vaccine. Here we report a case of CRS after vaccination with BNT162b2 (tozinameran), an mRNA COVID-19 vaccine.

**Fig. 1:**
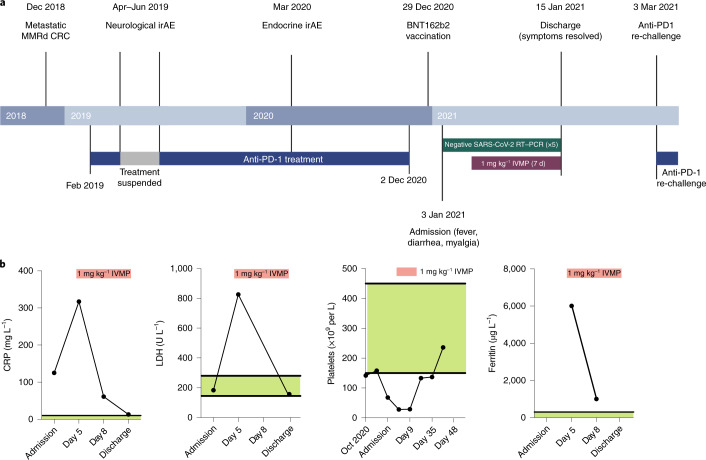
Clinical course from cancer diagnosis and inflammatory markers during CRS. **a**, Clinical timeline from diagnosis of metastatic MMRd CRC to CRS after one dose of BNT162b2 vaccine. Created with BioRender.com. **b**, CRP, LDH, platelet count and ferritin levels during the course of admission. Normal ranges are indicated in green. Treatment with IVMP is indicated in red.

A 58-year-old male commenced anti-PD-1 monotherapy (an investigational ICI within an ongoing interventional clinical trial; NCT02715284) in February 2019 for the treatment of mismatch repair-deficient colorectal cancer (MMRd CRC) metastatic to mesentry and rectus muscle (Fig. 1a). Two months after treatment initiation, he experienced a neurological immune-related adverse event (irAE) with worsening ataxia (grade 1 to grade 2, and magnetic resonance imaging changes in pons, medulla and cerebellum) on the background of pre-existing spinocerebellar ataxia of unknown etiology. ICI was suspended, and he was commenced on 1 mg kg^−1^ prednisolone (tapered over 1 month), and ataxia returned to grade 1 (baseline). Anti-PD-1 therapy was re-started in June 2019 (Fig. 1a), with stable disease as per immune-related Response Evaluation Criteria in Solid Tumors. In March 2020 (13 months after commencing ICI), he developed an endocrine irAE (grade 1 hypocortisolemia from adrenocorticotropic hormone deficiency; Fig. 1a) and was commenced on physiological corticosteroid replacement (prednisolone, 3 mg daily). Disease control was maintained, and the last ICI dose was administered in December 2020, 27 d before BNT162b2.

The patient had no history of severe acute respiratory syndrome coronavirus 2 (SARS-CoV-2) infection and had negative SARS-CoV-2 serological tests in June and October 2020. He received the first dose of BNT162b2 vaccine on 29 December 2020 (Fig. 1a) without immediate adverse events, except for grade 1 inflammation at the vaccination site. Five days later (32 d after the last anti-PD-1 dose), he presented with myalgia, 2-d history of diarrhea (grade 1) and 1-d history of fever (38.4 °C) despite anti-pyretics (ibuprofen) use. On admission to the hospital, his vital signs were as follows: oxygen saturation, 100% on room air; respiratory rate, 18 breaths per minute; blood pressure, 111/71 mmHg; heart rate, 86 beats per minute; and temperature, 36.7 °C. Laboratory investigations revealed elevated inflammatory markers (C-reactive protein (CRP)), 125 mg L^−1^ (normal, <6 mg L^−1^); serum lactate dehydrogenase (LDH), 184 U L^−1^ (normal range, 120–246 U L^−1^); and thrombocytopenia (68 × 10^9^ cells per liter (normal range, 150–410 cells per liter)), confirmed on microscopy (Fig. 1b). Empirical treatment with broad-spectrum intravenous antibiotics was commenced; however, blood and urine cultures were negative, as was SARS-CoV-2 RT–PCR of serial nasopharyngeal swabs (Fig. 1a). There were no clinical signs or symptoms during admission or follow-up to suspect a thrombotic event in this patient. Computed tomography of thorax, abdomen and pelvis revealed no nidus of infection or thrombosis and showed stable disease with respect to cancer. The patient was not heparinized. Over the next 5 d, fevers up to 39.8 °C continued, with worsening thrombocytopenia (28 × 10^9^ cells per liter) and increasing inflammatory markers (CRP, 317 mg L^−1^; LDH, 849 U L^−1^), including significantly elevated ferritin (6,010 µg L^−1^ (normal range, 18–464 µg L^−1^)) (Fig. 1b). At this point (5 d after admission), CRS was suspected (grade 3), and he was commenced on 1 mg kg^−1^ of intravenous methylprednisolone (IVMP), and antibiotics were ceased 3 d later. Biochemical and hematological indices normalized within 7 d of IVMP initiation (Fig. 1b), and the patient was afebrile and asymptomatic upon discharge home with a weaning corticosteroid regimen. He remained well and was re-challenged with anti-PD-1 on 8 February 2021 (36 d after initial presentation) without any adverse events (Fig. 1a). He did not receive the second dose of BNT162b2.

**Fig. 2:**
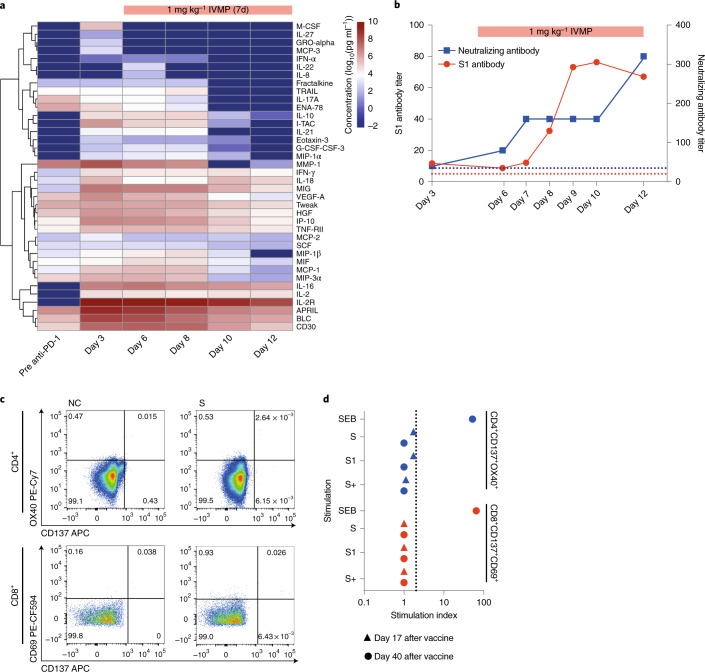
Cytokine profile and immune response to BNT162b2 vaccine. **a**, Cyto/chemokine levels were measured using the human immune monitoring 65-plex ProcartaPlex immunoassay in consecutive plasma samples. Samples were measured in duplicates. Data are presented as the log_10_ of the concentration in pg ml^−1^. **b**, Kinetics of S1-reactive and neutralizing antibody responses after BNT162b2. Data are presented as the reciprocal dilution of the last detected sample. IVMP treatment is indicated in red. **c**, SARS-CoV-2-specific CD4^+^ and CD8^+^ T cell response in exemplary samples after stimulation of PBMCs with spike (S) peptide pool. **d**, PBMCs were stimulated with S, S1 and S+ peptide pools representing the full length of the spike protein. SEB was used as a positive control. Data are presented as a stimulation index indicating the ratio of the frequency of CD4^+^CD137^+^OX40^+^ or CD8^+^CD137^+^CD69^+^ T cells in the sample and the negative control. SEB, Staphylococcal enterotoxin B.

To explore the features of his presentation further, we performed longitudinal cytokine analysis, before and after IVMP. An exaggerated type 1 helper T cell (Th1) response is a frequent feature of CRS^1^, and the initial profile (day 3 of admission; Fig. 2a) indicated activation of Th1 cells (elevated MIG, IL-2R, IL-16, IFN-γ and IL-18) and macrophages (elevated MCP-1, MIP, IL-8, IL-18 and MIG). IL-10 inhibits pro-inflammatory cytokines, limiting exuberant inflammatory responses^2^, and, although we observed elevated IL-10 on days 3–8 of admission, it evidently failed to suppress hyperinflammation in this case. Most cytokines decreased substantially during IVMP treatment, but persistent elevation of IL-2R, IL-2, IL-16 and IL-18 on day 12 of admission (Fig. 2a) indicated sustained T cell activation.

**Extended Data Fig. 1:**
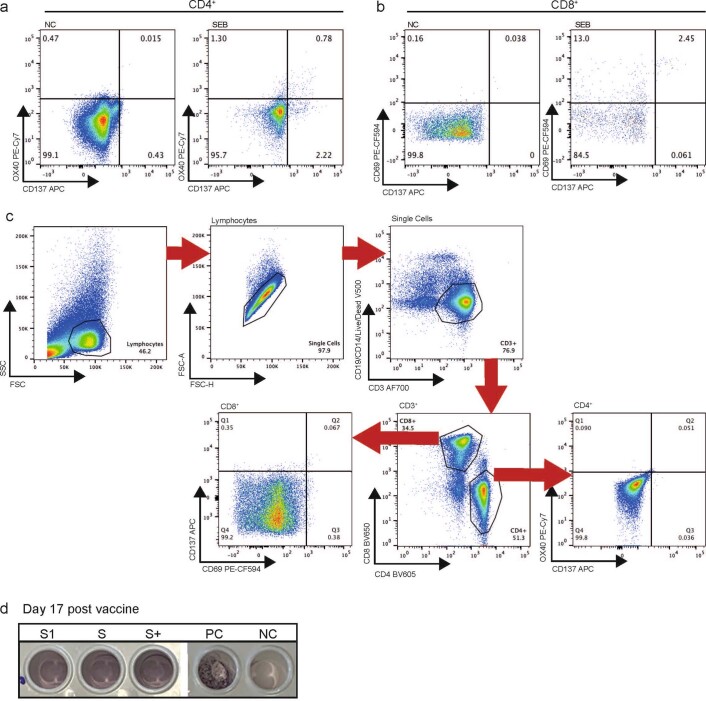
Gating strategy for T-cell assay. **a**, CD4 + T-cell response to SEB in exemplary samples. **b**, CD8 + T-cell response to SEB in exemplary samples. **c**, Gating strategy for flow analysis of T-cell activation. PBMCs were stimulated with SEB for 24 hrs. SEB, Staphylococcal enterotoxin B. **d**, IFN- γ ELISpot at day 17 post vaccine. PBMCs were stimulated with S, S1 and S + peptide pools representing the full length of the spike protein. Anti-CD3 antibody was used as a positive control (PC), no stimulation was added to the negative control (NC).

S1-reactive and neutralizing antibodies were detectable 7 d after vaccination (Fig. 2b), and the titers continued to rise during IVMP treatment, suggesting a robust vaccine-induced immune response. However, S-specific CD4^+^ and CD8^+^ T cells were undetectable on days 17 and 40 after vaccination, and no IFN-γ-producing T cells were detected (Fig. 2c,d,e and Extended Data Fig. 1a,b), consistent with reports in patients without cancer after the first dose of BNT162b2 (ref. ^5^). Data on the effect of steroids on mRNA vaccine-induced antigen-specific T cell response are limited. One study demonstrated that dexamethasone given before, but not after, an mRNA-based cancer vaccine resulted in reduced activation of antigen-specific T cells^6^. In the case under study, steroids were administered 10 d after vaccination and unlikely to have affected vaccine-specific immune response.

**Extended Data Fig. 2:**
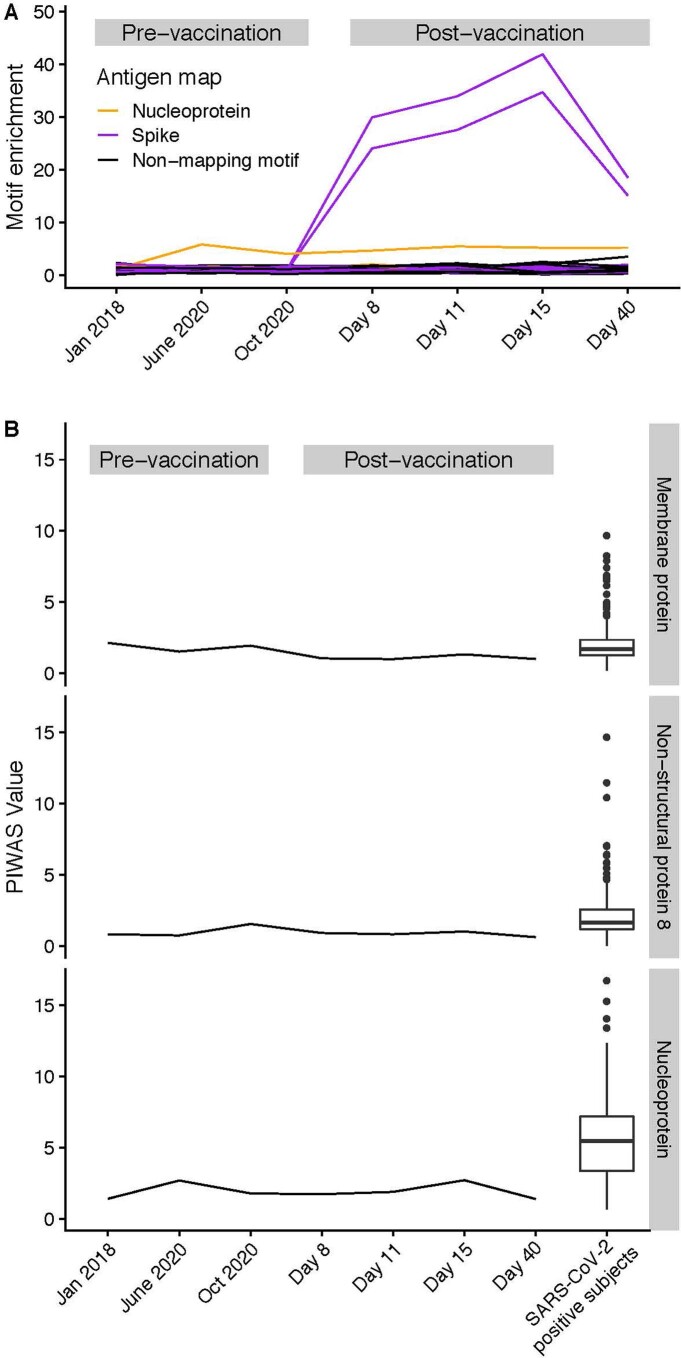
Antibody response to BNT162b2 according to SERA and PIWAS. **a**, Pre- and post-vaccination antibody levels against SARS-CoV-2 antigenic motifs. **b**, PIWAS values pre- and post-vaccination against non-spike sequences of the SARS-CoV-proteome compared to patients with SARS-CoV-2 positive subjects24 (Methods). Box plots of PIWAS values of SARS-CoV-2 positive subjects are shown, where boxes denote the IQR, and the median is shown as horizontal bars. Whiskers extend to 1.5x IQR and outliers are shown as individual points (n = 230 subjects; n = 230 biologically independent samples). IQR, interquartile range; PIWAS, protein-based immunome wide association study; SERA, serum epitope repertoire analysis.

To identify antibody-binding epitopes, we performed a serum epitope repertoire analysis (SERA) and a protein-based immunome-wide association study (PIWAS), using a bacterial display system coupled with next-generation sequencing. The post-vaccine profile was similar to that of healthcare workers after COVID-19 mRNA vaccine, with a rise in positive signal against spike but not non-spike proteins (versus patients positive for SARS-CoV-2)^7^ (Extended Data Fig. 2a,b). This was consistent with the lack of prior SARS-CoV-2 infection as a potential contributor to the clinical presentation.

**Extended Data Fig. 3:**
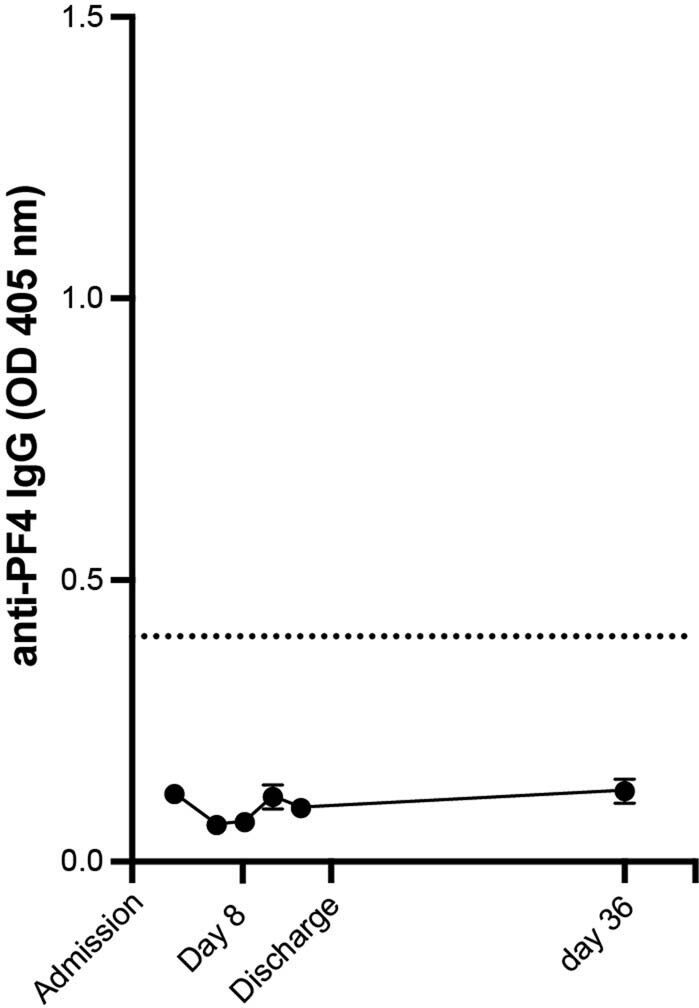
Anti-PF4 antibodies after vaccination. Serum samples collected after admission were analysed for presence of anti-PF4 antibodies by ELISA. Values represent mean of two technical replicates, error bars represent standard deviation. The dotted line indicates the threshold for a sample to be considered positive (OD 450 nm > 0.4).

Isolated thrombocytopenia (without thrombosis) has been described with mRNA-based COVID-19 vaccines, including BNT162b2 (ref. ^8^). Given recent reports of a pathogenic platelet factor-4 (PF4)-dependent syndrome leading to thrombotic thrombocytopenia after vaccination with ChAdOx1 nCov-19 (AstraZeneca)^9–11^, which is a viral vector-based COVID-19 vaccine, we evaluated for PF4 antibodies, which were not detectable (Extended Data Fig. 3). Although this result does not comprehensively exclude an independent mechanism for the observed thrombocytopenia, the constellation of clinical and laboratory findings make thrombocytopenia likely to be a component of CRS in this case.

The laboratory findings in CRS are variable and relate to the underlying cause, although CRP elevation is characteristic and correlates with severity^1^. Elevated ferritin and thrombocytopenia are also common abnormalities^1^. Although there are no defined cytokine profiles that confirm CRS, raised IFN-γ, IL-2R, IL-18, IL-6 and IL-10 are considered key in establishing the diagnosis^1,2^. All except IL-6 were elevated in this case. Although transient cytokine elevation (IFN-α, IFN-γ, IL-6, IFN-inducible protein-10 and IL-12p70) was observed after mRNA cancer vaccines co-administered with ICI in patients with melanoma^12^, they manifest as self-limiting mild flu-like symptoms.

Fewer than 0.01% of irAEs reported in the context of anti-PD-1 monotherapy involve CRS^4^, and, to date, no CRS events have been reported after either BNT162b2 or the mRNA-1273 vaccine (Moderna)—the two mRNA-based COVID-19 vaccines currently available. ICI-related CRS typically develops a median of 4 weeks after ICI initiation (range, 1–18 weeks)^4^, making ICI as the sole cause of CRS unlikely in this patient, who commenced anti-PD1 treatment 22 months prior. The close temporal association of vaccination and clinical presentation favors the vaccine as the potential trigger of CRS in this case.

Receptor occupancy associated with anti-PD-1 agents is 2–3 months^13^, and it remains possible that CRS was triggered by the vaccine on a background of immune activation secondary to PD1 blockade that results in T cell proliferation and increased effector function^14^. We did not detect S-reactive T cells in the periphery, and a direct mechanism for T cells driving CRS in this case could not be demonstrated. However, vaccine-activated T cells that contributed to CRS could be resident within tissue or lymph nodes and, therefore, undetectable in the blood^15^. T cell cross-reactivity, as a result of sequence similarity between spike protein and tumor neoantigens, is an alternative, although less likely, cause of CRS in this case. Cross-reactivity to cardiac tissue was reported as a mechanism of ICI-related myocarditis^16^, and this patient’s history of irAEs and the high neoantigen load (typical of MMRd CRC)^17^ could, in theory, increase the likelihood of T cell cross-reactivity^18^.

Given that patients with cancer were excluded from SARS-CoV-2 vaccine studies and are currently prioritized in COVID-19 vaccination programs globally, this case motivates prospective pharmacovigilance regarding the safety profile of COVID-19 vaccines in patients with cancer. So far, prospective data have not demonstrated additional safety concerns of BNT162b2 administration either in patients with cancer generally (*n* = 151)^19^ or, specifically, in those who have been treated with ICI (*n* = 170)^20^. Current empirical recommendations regarding the timing of COVID-19 vaccination suggest administering ‘on availability’ in patients with cancer on systemic anti-cancer treatments, including ICI, cytotoxic chemotherapy and hormone therapy, and avoiding vaccination within 48–72 h of investigational products to minimize misattribution of adverse event causation^21^. Overall, as CRS in this case is an isolated report, and patients with cancer are generally more vulnerable to COVID-19 (refs. ^22,23^), the benefit–risk profile for COVID-19 vaccination remains strongly in favor of vaccination in this population. It is of critical importance that patients with cancer remain prioritized during vaccine rollout^24^.

## Methods

### CAPTURE design, study schedule and follow-up

During admission, the patient was enrolled in CAPTURE (NCT03226886; see Supplementary Material for a list of consortium members), an observational prospective study of the immune response to SARS-CoV-2 in patients with cancer that opened for recruitment in May 2020 at the Royal Marsden NHS Foundation Trust. The study design was previously published^25^. Adult patients with current or history of invasive cancer are eligible for enrolment, irrespective of cancer type, stage or treatment. Primary and secondary endpoints relate to patient characteristics of those with and without SARS-CoV-2 infection and the effect of COVID-19 on long-term survival and intensive care unit admission rates. Exploratory endpoints pertain to characterizing clinical and immunological determinants of COVID-19 and vaccine response in patients with cancer. Clinical data and sample collection for participating patients with cancer are performed at baseline and at clinical visits per standard-of-care management during the first year of follow-up; frequency varies depending on inpatient or outpatient status and systemic anti-cancer treatment regimens. CAPTURE was approved as a substudy of TRACERx Renal (NCT03226886). TRACERx Renal was initially approved by the National Research Ethics Service (NRES) Committee London - Fulham on 17 January 2012. The TRACERx Renal sub-study CAPTURE was submitted as part of Substantial Amendment 9 and approved by the Health Research Authority on 30 April 2020 and the NRES Committee London - Fulham on 1 May 2020. The CAPTURE protocol was approved by institutional review boards and ethics committees, and the participant in this case report gave written informed consent for sample collection and use, according to CARE guidelines and in compliance with Declaration of Helsinki principles.

### Adverse events grading

All adverse events were graded per Common Terminology Criteria for Adverse Events version 4.03.

### Handling of whole blood samples

For indicated experiments, serum or plasma samples were heat inactivated at 56 °C for 30 min before use.

### Plasma and peripheral blood mononuclear cell isolation

Whole blood was collected in EDTA tubes (VWR) and stored at 4 °C until processing. All samples were processed within 24 h. Time of blood draw, processing and freezing was recorded for each sample. Before processing, tubes were brought to room temperature. Peripheral blood mononuclear cells (PBMCs) and plasma were isolated by density gradient centrifugation using pre-filled centrifugation tubes (pluriSelect). Up to 30 ml of undiluted blood was added on top of the sponge and centrifuged for 30 min at 1,000*g* at room temperature. Plasma was carefully removed and then centrifuged for 10 min at 4,000*g* to remove debris, aliquoted and stored at −80 °C. The cell layer was then collected and washed twice in PBS by centrifugation for 10 min at 300*g* at room temperature. PBMCs were resuspended in Recovery Cell Culture Freezing Medium (Thermo Fisher Scientific) containing 10% DMSO, placed overnight in CoolCell freezing containers (Corning) at −80 °C and then stored in liquid nitrogen.

### Serum isolation

Whole blood was collected in serum coagulation tubes (Vacuette CAT tubes, Greiner Bio-One) for serum isolation and stored at 4 °C until processing. All samples were processed within 24 h. Time of blood draw, processing and freezing was recorded for each sample. Tubes were centrifuged for 10 min at 2,000*g* at 4 °C. Serum was separated from the clotted portion, aliquoted and stored at −80 °C.

### S1-reactive IgG ELISA

Ninety-six-well MaxiSorp plates (Thermo Fisher Scientific) were coated overnight at 4 °C with purified S1 protein in PBS (3 μg ml^−1^ per well in 50 μl) and blocked for 1 h in blocking buffer (PBS, 5% milk, 0.05% Tween 20 and 0.01% sodium azide). Sera were diluted in blocking buffer (1:50). Fifty microliters of serum was then added to the wells and incubated for 2 h at room temperature. After washing four times with PBS-T (PBS and 0.05% Tween 20), plates were incubated with alkaline phosphatase-conjugated goat anti-human IgG (1:1,000, Jackson ImmunoResearch) for 1 h. Plates were developed by adding 50 μl of alkaline phosphatase substrate (Sigma-Aldrich) for 15–30 min after six washes with PBS-T. Optical densities were measured at 405 nm on a microplate reader (Tecan). CR3022 (Absolute Antibody) was used as a positive control. The cutoff for a positive response was defined as the mean negative value multiplied by 0.35 times the mean positive value.

### Neutralizing antibody assay

Confluent monolayers of Vero E6 cells were incubated with SARS-CoV-2 virus and two-fold serial dilutions of heat-treated serum or plasma samples starting at 1:40 for 4 h at 37 °C in 5% CO_2_ in duplicates. The inoculum was then removed, and cells were overlaid with viral growth medium. Cells were incubated at 37 °C in 5% CO_2_. At 24 h after infection, cells were fixed in 4% paraformaldehyde (PFA) and permeabilized with 0.2% Triton X-100/PBS. Virus plaques were visualized by immunostaining, as described previously^26^ for the neutralization of influenza viruses using a rabbit polyclonal anti-NSP8 antibody used at 1:1,000 dilution and anti-rabbit horseradish peroxidase (HRP)-conjugated antibody at 1:1,000 dilution and detected by action of HRP on a tetramethyl benzidine-based substrate. Virus plaques were quantified, and ID_50_ was calculated.

### T cell stimulation

PBMCs for in vitro stimulation were thawed at 37 °C and resuspended in 10 ml of warm complete medium (RPMI and 5% human AB serum) containing 0.02% benzonase. Viable cells were counted, and 2 × 10^6^ cells were seeded in 200 µl of complete medium per well of a 96-well plate. Cells were stimulated with 4 µl per well of PepTivator SARS-CoV-2 S, M, or N pools (representing 1 µg ml^−1^ final concentration per peptide; Miltenyi Biotec). Staphylococcal enterotoxin B (Merck) was used as a positive control at 0.5 µg ml^−1^ final concentration; negative control was PBS containing DMSO at 0.002% final concentration. PBMCs were cultured for 24 h at 37 °C in 5% CO_2_.

### Activation-induced marker assay

Cells were washed twice in warm PBMCs. Dead cells were stained with 0.5 µl per well of Zombie dye V500 for 15 min at room temperature in the dark and then washed once with PBS containing 2% FCS (FACS buffer). A surface staining mix was prepared per well, containing 2 µl per well of each antibody for surface staining (Supplementary Table 1) in 50:50 brilliant stain buffer (BD Biosciences) and FACS buffer. PBMCs were stained with 50 µl of surface staining mix per well for 30 min at room temperature in the dark. Cells were washed once in FACS buffer and fixed in 1% PFA in FACS buffer for 20 min and then washed once and resuspended in 200 µl of PBS. All samples were acquired on a Bio-Rad ZE5 flow cytometer running Bio-Rad Everest software version 2.4 and analyzed using FlowJo version 10 (Tree Star) analysis software. Compensation was performed with 20 µl of antibody-stained Anti-Mouse Ig, k/Negative Control Compensation Particles Set (BD Biosciences). Up to 1 × 10^6^ live CD19^−^CD14^−^ cells were acquired per sample. Gates were drawn relative to the unstimulated control for each donor. Gating strategy is shown in Supplementary Fig. 2c. T cell response is displayed as a stimulation index by dividing the percentage of apoptosis inhibitor of macrophage (AIM)-positive cells by the percentage of cells in the negative control. When S, M and N stimulation were combined, the sum of AIM-positive cells was divided by the three times the percentage of positive cells in the negative control.

### ELISpot assay

IFN-γ pre-coated ELISpot plates (Mabtech) were blocked with complete medium (RPMI and 5% human AB serum) before 300,000 PBMCs were seeded per well and stimulated for 18 h with 2 µl per well of PepTivator SARS-CoV-2 S, M or N pools (representing 1 µg ml^−1^ final concentration per peptide; Miltenyi Biotec). Plates were developed with human biotinylated IFN-γ detection antibody (7-B6-1-ALP, 1:200), followed by incubation with BCIP/NBT Phosphatase Substrate (SeraCare). Spot-forming units (SFU) were quantified with ImmunoSpot (Mabtech). To quantify positive peptide-specific responses, spots of the unstimulated wells were subtracted from the peptide-stimulated wells, and the results were expressed as SFU/106 PBMCs.

### Multiplex immune assay for cytokines and chemokines

The pre-configured multiplex Human Immune Monitoring 65-plex ProcartaPlex immunoassay kit (Invitrogen, Thermo Fisher Scientific) was used to measure 65 protein targets in plasma on the Bio-Plex platform (Bio-Rad Laboratories), using Luminex xMAP technology. Analytes measured included APRIL; BAFF; BLC; CD30; CD40L; ENA-78; eotaxin; eotaxin-2; eotaxin-3; FGF-2; fractalkine; G-CSF; GM-CSF; GRO-alpha; HGF; IFN-α; IFN-γ; IL-10; IL-12p70; IL-13; IL-15; IL-16; IL-17A; IL-18; IL-1α; IL-1β; IL-2; IL-20; IL-21; IL-22; IL-23; IL-27; IL-2R; IL-3; IL-31; IL-4; IL-5; IL-6; IL-7; IL-8; IL-9; IP-10; I-TAC; LIF; MCP-1; MCP-2; MCP-3; M-CSF; MDC; MIF; MIG; MIP-1α; MIP-1β; MIP-3α; MMP-1; NGF-β; SCF; SDF-1α; TNF-β; TNF-α; TNF-R2; TRAIL; TSLP; TWEAK; and VEGF-A. All assays were conducted as per manufacturer recommendations.

### SERA

Patient serum samples were screened and analyzed using the previously published SERA pipeline^27^. Briefly, sera were screened with a randomized bacterial peptide display library, and plasmids from antibody-bound bacteria were isolated and sequenced. PIWAS was applied to identify epitopes and antigens for the SARS-CoV-2 proteome.

### PF-4 IgG assay

Patient serum samples were analyzed using the LIFECODES PF-4 IgG Solid Phase ELISA microwells assay (Immucor). Briefly, diluted serum and controls were added to microwells coated with PF-4 complexed to polyvinyl sulfonate, incubated for 45 min at 37 °C and washed. IgG conjugate was added and incubated for 45 min at 37 °C. Plates were developed by incubation PNPP solution. Absorbance of each well was read at 405 nm. A positive and negative serum control was measured on the same plate. Values greater than 0.4 were considered positive.

### Reporting Summary

Further information on research design is available in the Nature Research Reporting Summary linked to this article.

## Online content

Any methods, additional references, Nature Research reporting summaries, source data, extended data, supplementary information, acknowledgements, peer review information; details of author contributions and competing interests; and statements of data and code availability are available at 10.1038/s41591-021-01387-6.

## Supplementary information

Supplementary Information Supplementary Table 1 and lists of CAPTURE and Crick COVID-19 consortia members.

Reporting Summary

## Data Availability

All requests for raw and analyzed data, materials and CAPTURE study protocol will be reviewed by the CAPTURE Trials Team, Skin and Renal Clinical Trials Unit, The Royal Marsden NHS Foundation Trust (CAPTURE@rmh.nhs.uk) to determine whether the request is subject to confidentiality and data protection obligations. Data and materials that can be shared will be released via a material transfer agreement.
