# Supplementary material for: Cytokine release syndrome in a patient with colorectal cancer after vaccination with BNT162b2
Source: Nat Med. 2021 May 26;27(8):1362–6. doi: 10.1038/s41591-021-01387-6 (PMC8363501; doi:10.1038/s41591-021-01387-6)
Supplement: Supplementary file 1 — Supplementary Table 1 and lists of CAPTURE and Crick COVID-19 consortia members. [file 41591_2021_1387_MOESM1_ESM.pdf]

nature portfolio

https://doi.org/10.1038/s41591-021-01387-6

Supplementary information

Cytokine release syndrome in a patient with colorectal cancer after vaccination with BNT162b2

In the format provided by the authors and unedited

Supplementary Tables

Table S1: List of antibodies used in AIM assay

Antibody	Fluorochrome	Clone	Dilution	Lot-No	Vendor	Cat-No.
**CD14**	V500	M5E2	1:25	0282746	BD	561391
**CD19**	V500	HIB19	1:25	1018553	BD	561121
**CD4**	BV605	OKT4	1:25	B327902	Biolegend	317438
**CD8**	BV650	RPA-T8	1:25	B323717	Biolegend	301042
**CD69**	PE-CF594	FN50	1:25	0337206	BD	562617
**OX40**	PE-Cy7	Ber-ACT35	1:25	B326195	Biolegend	350012
**CD137**	APC	4B4-1	1:25	B320971	Biolegend	309810
**CD3**	AF700	OKT3	1:25	317340	Biolegend	B279661
**NSP8**	NA	polyclonal	1:1000	17040	antibodies-online	ABIN233792
**goat anti-rabbit IgG HRP**	NA	NA	1:1000	NA	Biorad	170-6515
**7-B6-ALP**	NA		1:200	393	Mabtech	3420-2APT-10
**CD3-2**	NA	NA	1:1000	393	Mabtech	3605-1-S

The Crick COVID-19 Consortium

Titilayo Abiola, Jim Aitken, Zoe Allen, Rachel Ambler, Karen Ambrose, Emma Ashton, Alida Avola, Samutheswari Balakrishnan, Caitlin Barns-Jenkins, Genevieve Barr, Sam Barrell, Souradeep Basu, Rodrigo Batalha, Rupert Beale, Clare Beesley, Teresa Bertran, Natalie Bevan, Nisha Bhardwaj, Shahnaz Bibi, Ganka Bineva-Todd, Dhruva Biswas, Michael J Blackman, Dominique Bonnet, Carles Bosch, Faye Bowker, Malgorzata Broncel, Claire Brooks, Michael D Buck, Andrew Buckton, Timothy Budd, Alana Burrell, Louise Busby, Claudio Bussi, Simon Butterworth, Matthew Byott, Fiona Byrne, Richard Byrne, Simon Caidan, Veronique Calleja, Enrica Calvani, Joanna Campbell, Johnathan Canton, Ana Cardoso, Nick Carter, Luiz Carvalho, Raffaella Carzaniga, Antonio Casal, Natalie Chandler, Qu Chen, Peter Cherepanov, Laura Churchward, Graham Clark, Bobbi Clayton, Clementina Cobolli Gigli, Zena Collins, Nicola Cook, Cristina Cotobal Martin, Sally Cottrell, Margaret Crawford, Stefania Crotta, Laura Cubitt, Tom Cullup, Annalisa D'Avola, Heledd Davies, Patrick Davis, Dara Davison, Joost De Folter, Vicky Dearing, Solene Debaisieux, Monica Diaz-Romero, Alison Dibbs, Jessica Diring, Paul C Driscoll, Christopher Earl, Amelia Edwards, Chris Ekin, Dimitrios Evangelopoulos, Todd Fallesen, Rupert Faraway, Antony Fearns, Aaron Ferron, Efthymios Fidanis, Patricia Figuiredo-Nunes, Katja Finsterbusch, Dan Fitz, James Fleming, Helen Flynn, Ashley Fowler, Daniel Frampton, Bruno Frederico, Alessandra Gaiba, Anthony Gait, Steve Gamblin, Sonia Gandhi, Julian Gannon, Edmund Garr, Kathleen Gärtner, Acely Garza-Garcia, Liam Gaul, Helen M Golding, Jacki Goldman, Robert Goldstone, Belen

Gomez Dominguez, Hui Gong, Ilaria Gori, Paul R Grant, Maria Greco, Mariana Grobler, Anabel Guedan, Silvana Guioli, Maximiliano G Gutierrez, Fiona Hackett, Chris Hadjigeorgiou, Ross Hall, Steinar Halldorsson, Suzanne Harris, Sugera Hashim, Emine Hatipoglu, Lyn Healy, Judith Heaney, Susanne Herbst, Graeme Hewitt, Theresa Higgins, Prisca Hill, Steve Hindmarsh, Rajnika Hirani, Han Ngoc Ho, Maxine Holder, Joshua Hope, Elizabeth Horton, Beth Hoskins, Catherine F Houlihan, Michael Howell, Louise Howitt, Jacqueline Hoyle, Mint R Htun, Michael Hubank, Hector Huerga Encabo, Deborah Hughes, Jane Hughes, Almaz Huseynova, Ming-Shih Hwang, Fairouz Ibrahim, Rachael Instrell, Deborah Jackson, Mariam Jamal-Hanjani, Lucy Jenkins, Ming Jiang, Mark Johnson, Leigh Jones, Neil Justin, Nnennaya Kanu, George Kassiotis, Gavin Kelly, Geoff Kelly, Louise Kiely, Anastacio King Spert Teixeira, Fiona Kinnis, Stuart Kirk, Svend Kjaer, Ellen Knuepfer, Nikita Komarov,Paul Kotzampaltiris, Konstantinos Kousis,Tammy Krylova, Ania Kucharska, Robyn Labrum, Catherine Lambe, Michelle Lappin, Stacey-Ann Lee, Andrew Levett, Lisa Levett, Marcel Levi, Nick Lewis, Hon-Wing Liu, Shuangyan Liu, Sam Loughlin, Wei-Ting Lu, Robert Ludwig, James I MacRae, Akshay Madoo, Sarah Manni, Julie A Marczak, Manuella Marques, Mimmi Martensson, Thomas Martinez, Bishara Marzook, John Matthews, Joachim M Matz, Samuel McCall, Laura E McCoy, Fiona McKay, Edel C McNamara, Sofanit Mebrate, Hilina Mehari, Manuela Melchionda, Carlos M Minutti, Gita Mistry, Miriam Molina-Arcas, Beatriz Montaner, Kylie Montgomery, Catherine Moore, David Moore, Anastasia Moraiti, Raveena Morar, Lucia Moreira-Teixeira, Joyita Mukherjee, Cristina Naceur-Lombardelli, Eleni Nastouli, Aileen Nelson, Jerome Nicod, Luke Nightingale, Stephanie Nofal, Paul Nurse, Savita Nutan, Anne O'Garra, Jean D O'Leary, Olga O'Neill, Nicola O'Reilly, Caroline Oedekoven, Jessica Olsen, Paula Ordonez Suarez, Neil Osborne, Amar Pabari, Aleksandra Pajak, Stavroula M Paraskevopoulou, Namita Patel, Yogen Patel, Oana Paun, Nigel Peat, Laura Peces-Barba Castano, Ana Perez Caballero, Jimena Perez-Lloret, Magali S Perrault, Abigail Perrin, Roy Poh, Enzo Z Poirier, James M Polke, Marc Pollitt, Lucia Prieto-Godino, Alize Proust, Clinda Puvirajasinghe, Val Pye, Christophe Queval, Vijaya Ramachandran, Abhinay Ramaprasad, Peter Ratcliffe, Minoo Razi, Laura Reed, Caetano Reis e Sousa, Kayleigh Richardson, Sophie Ridewood, Karine Rizzoti, Fiona Roberts, Rowenna Roberts, Angela Rodgers, Pablo Romero Clavijo, Annachiara Rosa, Alice Rossi, Chloe Roustan, Andrew Rowan, Erik Sahai, Aaron Sait,
Katarzyna Sala, Emilie Sanchez, Theo Sanderson, Pierre Santucci, Fatima Sardar, Adam Sateriale, Jill A Saunders, Chelsea Sawyer, Anja Schlott, Edina Schweighoffer, Sandra Segura-Bayona, Rajvee Shah Punatar, Maryam Shahmanesh, Joe Shaw, Gee Yen Shin, Mariana Silva Dos Santos, Margaux Silvestre, Matthew Singer, Marie Sjothun, Daniel M Snell, Ok-Ryul Song, Christelle Soudy, Moira J Spyer, Louisa Steel, Amy Strange, Adrienne E Sullivan, Charles Swanton, Michele SY Tan, Zoe H Tautz-Davis, Raquel Taveira-Marques, Effie Taylor, Gunes Taylor, Harriet B Taylor, Alison Taylor-Beadling, Fernanda Teixeira Subtil, Berta Terré, Goran Tomic, Patrick Toolan-Kerr, Francesca Torelli, Tea Toteva, Moritz

Treeck, Hadija Trojer, Ming-Han C Tsai, James MA Turner, Melanie Turner, Jernej Ule, Rachel Ulferts, Sharon P Vanloo, Selvaraju Veeriah, Subramanian Venkatesan, Ferdinando Verdirame, Karen Vousden, Andreas Wack, Claire Walder, Jane Walker, Philip A Walker, Yiran Wang, Sophia Ward, Catharina Wenman, Luke Wiliams, Matthew J Williams, Cherry Wong, Wai Keong Wong, Chi Wong, Joshua Wright, Mary Wu, Lauren Wynne, Zheng Xiang, Melvyn Yap, Julian A Zagalak, Davide Zecchin, Rachel Zillwood.

The CAPTURE consortium

Lewis Au, Susana Banerjee, Katie Bentley, Shree Bhide, Laura Amanda Boos, Fiona Byrne, Ian Chau, David Cunningham, Joanne Droney, Annika Fendler, Andrew J.S. Furness, Camille Gerard, Firza Gronthud, Kevin Harrington, Adrian Hayday, Shaman Jhanji, Robin Jones, George Kassiotis, Yasir Khan, Sacheen Kumar, James Larkin, Steve K.W. Leung, Michael Jones, Paula Lorgelly, Richard Martin, Ethna McFerran, Christina Messiou, Emma Nicholson, Alicia Okines, Clare Peckitt, Lisa Pickering, Karin Purshouse, Alison Reid, Caroline Relton, Jennifer Rusby, Scott T.C. Shepherd, Ben Shum, Tim Slattery, Naureen Starling, Anthony Swerdlow, Stefan Symeonides, Kate Tatham, Samra Turajlic, Nicholas Turner, Liam Welsh, Katalin Wilkinson, Robert J Wilkinson, Matthew Wheater, Kate Young, and Nadia Yousaf.
