# Supplementary material for: Cytokine release syndrome in a patient with colorectal cancer after vaccination with BNT162b2
Source: Nat Med. 2021 May 26;27(8):1362–6. doi: 10.1038/s41591-021-01387-6 (PMC8363501; doi:10.1038/s41591-021-01387-6)
Supplement: Supplementary file 2 — Reporting Summary [file 41591_2021_1387_MOESM2_ESM.pdf]

nature research

Corresponding author(s): Samra Turajlic

Last updated by author(s): 20/03/21

Reporting Summary

Nature Research wishes to improve the reproducibility of the work that we publish. This form provides structure for consistency and transparency in reporting. For further information on Nature Research policies, see our Editorial Policies and the Editorial Policy Checklist.

Statistics

For all statistical analyses, confirm that the following items are present in the figure legend, table legend, main text, or Methods section.

n/a	Confirmed	
☐	☒	The exact sample size (*n*) for each experimental group/condition, given as a discrete number and unit of measurement
☐	☒	A statement on whether measurements were taken from distinct samples or whether the same sample was measured repeatedly
☒	☐	The statistical test(s) used AND whether they are one- or two-sided<br>*Only common tests should be described solely by name; describe more complex techniques in the Methods section.*
☒	☐	A description of all covariates tested
☒	☐	A description of any assumptions or corrections, such as tests of normality and adjustment for multiple comparisons
☒	☐	A full description of the statistical parameters including central tendency (e.g. means) or other basic estimates (e.g. regression coefficient) AND variation (e.g. standard deviation) or associated estimates of uncertainty (e.g. confidence intervals)
☒	☐	For null hypothesis testing, the test statistic (e.g. *F*, *t*, *r*) with confidence intervals, effect sizes, degrees of freedom and *P* value noted<br>*Give P values as exact values whenever suitable.*
☒	☐	For Bayesian analysis, information on the choice of priors and Markov chain Monte Carlo settings
☒	☐	For hierarchical and complex designs, identification of the appropriate level for tests and full reporting of outcomes
☒	☐	Estimates of effect sizes (e.g. Cohen's *d*, Pearson's *r*), indicating how they were calculated

Our web collection on statistics for biologists contains articles on many of the points above.

Software and code

Policy information about availability of computer code

Data collection	N/A
Data analysis	All statistical analyses were performed in FlowJo v10.7.1 and GraphPad Prism v9.1.0

For manuscripts utilizing custom algorithms or software that are central to the research but not yet described in published literature, software must be made available to editors and reviewers. We strongly encourage code deposition in a community repository (e.g. GitHub). See the Nature Research guidelines for submitting code & software for further information.

Data

Policy information about availability of data

All manuscripts must include a data availability statement. This statement should provide the following information, where applicable:

- Accession codes, unique identifiers, or web links for publicly available datasets
- A list of figures that have associated raw data
- A description of any restrictions on data availability

All requests for raw and analysed data, materials, and CAPTURE study protocol will be reviewed by the CAPTURE Trials Team, Skin and Renal Clinical Trials Unit, The Royal Marsden NHS Foundation Trust (CAPTURE@rmh.nhs.uk) to determine if the request is subject to confidentiality and data protection obligations. Data and materials that can be shared will be released via a material transfer agreement.

Field-specific reporting

Please select the one below that is the best fit for your research. If you are not sure, read the appropriate sections before making your selection.

☒ Life sciences ☐ Behavioural & social sciences ☐ Ecological, evolutionary & environmental sciences

For a reference copy of the document with all sections, see nature.com/documents/nr-reporting-summary-flat.pdf

Life sciences study design

All studies must disclose on these points even when the disclosure is negative.

Sample size	This is a case report (n=1).
Data exclusions	No data were excluded.
Replication	For correlative measures, all human specimens underwent quality control (QC) assessments. Only those that passed QC were further analyzed
Randomization	This is a case report (n=1).
Blinding	This is a case report and thus blinding was not performed.

Reporting for specific materials, systems and methods

We require information from authors about some types of materials, experimental systems and methods used in many studies. Here, indicate whether each material, system or method listed is relevant to your study. If you are not sure if a list item applies to your research, read the appropriate section before selecting a response.

Materials & experimental systems

n/a	Involved in the study	
☐	☒	Antibodies
☒	☐	Eukaryotic cell lines
☒	☐	Palaeontology and archaeology
☒	☐	Animals and other organisms
☐	☒	Human research participants
☐	☒	Clinical data
☒	☐	Dual use research of concern

Methods

n/a	Involved in the study	
☒	☐	ChIP-seq
☐	☒	Flow cytometry
☒	☐	MRI-based neuroimaging

Antibodies

Antibodies used	A list of antibodies is provided in Table S1
Validation	Antibodies for AIM assay were chosen on the basis of previous publication of the assay (Grifoni et al. Targets of T Cell Responses to SARS-CoV-2 Coronavirus in Humans with COVID-19 Disease and Unexposed Individuals. Cell.) References with validation of other primary antibodies used are as follows: Neutralising antibodies (NSP8 and anti-rabbit IgG - Wrobel et al. Antibodymediated disruption of the SARS-CoV-2 spike glycoprotein. Nat Comm.; ELISpot assay antibodies (https://www.mabtech.com/products/human-ifn-gamma-elisa-pro-kit_3420-1hp-10).

Human research participants

Policy information about studies involving human research participants

Population characteristics	Single human subject. 58 year old male with history of metastatic mismatch repair deficient colorectal cancer on anti-PD1 monotherapy.
Recruitment	CAPTURE study (NCT03226886)
Ethics oversight	CAPTURE was approved as a substudy of TRACERx Renal (NCT03226886). TRACERx Renal was initially approved by the NRES Committee London - Fulham on January 17, 2012. The TRACERx Renal sub-study CAPTURE was submitted as part of Substantial Amendment 9 and approved by the Health Research Authority on April 30, 2020 and the NRES Committee London - Fulham on May 1, 2020. CAPTURE is conducted in accordance with the ethical principles of the Declaration of Helsinki, Good Clinical Practice and applicable regulatory requirements.

Note that full information on the approval of the study protocol must also be provided in the manuscript.

Clinical data

Policy information about clinical studies

All manuscripts should comply with the ICMJE guidelines for publication of clinical research and a completed CONSORT checklist must be included with all submissions.

Clinical trial registration	CAPTURE study (NCT03226886)
Study protocol	Request for protocols should be directed to CAPTURE trials unit via CAPTURE@rmh.nhs.uk
Data collection	Data was collected at Royal Marsden hospital hospital, by extract from clinical records approved as per protocol. The participant was recruited on the 13/1/21.
Outcomes	Vaccine efficacy, immunological parameters, and associations with clinical features presented are exploratory endpoints of this study. Primary endpoint of study is description of population characteristics between SARS-CoV-2 positive and negative cancer patients. Secondary endpoints are differences in overall survival, intensive treatment unit admission rate, anti-cancer treatment received, and immune related adverse events.

Flow Cytometry

Plots

Confirm that:

- [x] The axis labels state the marker and fluorochrome used (e.g. CD4-FITC).
- [x] The axis scales are clearly visible. Include numbers along axes only for bottom left plot of group (a 'group' is an analysis of identical markers).
- [x] All plots are contour plots with outliers or pseudocolor plots.
- [x] A numerical value for number of cells or percentage (with statistics) is provided.

Methodology

Sample preparation	Whole blood was collected in EDTA tubes (VWR) and stored at 4ºC until processing. All samples were processed within 24 hours. Time of blood draw, processing, and freezing was recorded for each sample. Prior to processing tubes were brought to room temperature (RT). PBMC and plasma were isolated by density-gradient centrifugation using pre-filled centrifugation tubes (pluriSelect). Up to 30 ml of undiluted blood was added on top of the sponge and centrifuged for 30 minutes at 1000 x g at RT. Plasma was carefully removed then centrifuged for 10 minutes at 4000 x g to remove debris, aliquoted and stored at -80ºC. The cell layer was then collected and washed twice in PBS by centrifugation for 10 minutes at 300 x g at RT. PBMC were resuspended in Recovery cell culture freezing medium (Fisher Scientific) containing 10% DMSO.
Instrument	All experiments were run on a Bio-Rad Ze5 flow cytometer running Bio-Rad Everest software v2.4
Software	Data were analysed using FlowJo 10.7.1
Cell population abundance	Cells were not sorted in this study
Gating strategy	Lymphocytes were gated in FSC-A/SSC-A plot, followed by gating for singlets by plotting FSC-A vs. FSC-H. Viable CD3+ cells were identified by plotting CD3 vs. CD14,CD19, and viability dye. Next CD4+ and CD4+ cells were gated and finally CD137 +OX40+ cells were identified in the CD4+ population and CD137+CD69+ in the CD8+ population.

- [x] Tick this box to confirm that a figure exemplifying the gating strategy is provided in the Supplementary Information.
